# Phytochemical Analysis, Antioxidant and Antibacterial Properties of *Spilanthes mauritiana* Used Traditionally in Limpopo Province, South Africa

**DOI:** 10.1177/2515690X17746774

**Published:** 2017-12-17

**Authors:** Peter Masoko

**Affiliations:** 1 University of Limpopo, Sovenga, South Africa

**Keywords:** *Spilanthes mauritiana*, antibacterial, antioxidant, phytochemical analysis

## Abstract

*Spilanthes mauritiana* belonging to the family Asteraceae, was screened for biological activity against bacterial pathogen. Antibacterial activity of the plant was investigated using microbroth dilution assay and bioautography. Total phenols and tannins of the extract were 52.47 ± 2.29 and 23.9 ± 1.18 as mg of gallic acid equivalents, respectively. Total flavonoid content was 25.1 ± 0.79 as mg of quercetin equivalents. Free radical scavenging activity of constituents in all the extract, against 2,2-diphenyl-1-picrylhydrazyl showed minimal activity. All extracts contained compounds with antibacterial activity against tested pathogens. Hexane extract had an average minimum inhibitory concentration value of 2.50 mg/mL, followed by methanol extract (1.72 mg/mL), acetone and dichloromethane extracts (1.96 mg/mL). The present study revealed the presence of compounds in *S mauritiana* with potent antibacterial activity against tested pathogens that are exhibiting the observed activity independent of other constituents contained in the extracts.

Plants have been used as a source of medicine throughout the world to preserve human health and are rich source components with a variety of biological activities, including antibacterial activity. Secondary metabolites contained in plants are responsible for their medicinal activities.^1^ Approximately 95% of modern drugs have been isolated from traditional medicinal plants.^2^

In most cases, these plants are traditionally used in the form of infusion, decoction, tincture, or herbal extracts. In some other cases, they are used in combination with 2 or more plants for treatment of a particular disease.^3^ Recently, pathogenic microorganisms have acquired resistance genes toward currently available antibiotics used in health care systems. There is therefore a great need to screen medicinal plants for potential and effective antibacterial drugs against resistance strains.^4^ On the other hand, oxygen plays an important role in biological systems of aerobic forms of life, although its derivatives are highly toxic. Free radicals are produced either as by-products or end products of some biochemical reactions that contribute to the development and maintenance of cells. High levels of free radicals in normal systems result in imbalanced between antioxidants and free radicals that leads to various diseases, including cancer.^5^ Medicinal plants are a rich source of antioxidant compounds. Antioxidants play an important role in the neutralization, inactivation, and scavenging of free radicals or reactive oxygen species (ROS) to reduce the risk of degenerative and chronic diseases.^6^

*Spilanthes mauritiana* (Asteraceae) is locally known as Tshishengelaphofu by the Tshivenda speaking tribe of South Africa. The leaves and flowers of *Spilanthes* spp are also called toothache herb. The plant has been traditionally used as medicines in South Africa, Cameroon, and Germany for treatment of toothache, stomatitis, throat complaints, malaria, soreness, dysentery, and gum care. The leaves are chewed in the form of a tincture to treat gum diseases and throat infections.^7,8^ *S mauritiana* has been reported for its biological activities such as insecticides and larvicidal properties^9^ and nonactivity against *Streptococcus pyogenes*.^7^

There are several methods used in medical research laboratories to test for bioactive compounds with antimicrobial activity, which includes bioautography and microbroth dilution methods.^10^ The aim of the study was to investigate the phytoconstituents, antibacterial and antioxidant activity of *S mauritiana*.

## Methods and Materials

### Plant Collection

Plant was collected in summer of 2014 from Luheni area, Dzimauli, Limpopo, South Africa. The voucher specimen was deposited to Larry Leach Herbarium (UNIN) for confirmation. Voucher specimens (UNIN 12294) was identified, verified, and substantiated by Dr Brownyn Egan. The plant was selected based on the traditional knowledge of its use in the Dzimauli area, Limpopo Province, South Africa.

### Plant Storage

The whole fresh plant was dried at room temperature. Most scientists have tended to use dried plant material because there are fewer problems associated with large-scale extraction of dried plants.^11^ The whole plant was milled to fine powder using blender and stored at room temperature in closed bottles in the dark until extracted.

### Extraction Procedure

Crude extracts were prepared by weighing 1.0 g of finely ground plant material and macerate in 10 mL of *n*-hexane, dichloromethane, acetone, and methanol (technical grade-Merck). The tubes were vigorously shaken for 10 minutes at 300 rpm. Plant residues were allowed to settle and the supernatant was filtered using a Whatman No. 3 filter into preweighed glass beakers. The process was repeated 3 times to exhaustively extract the plant material and the extracts from each solvent were combined. The solvent was removed under a stream of cold air at a room temperature. Plant extracts were reconstituted in acetone to a final concentration of 10 mg/mL.

### Analysis of Extracts by Thin Layer Chromatography

Ten microliters of 10 mg/mL extracts were loaded onto a thin layer chromatography (TLC) plates. Three separation systems of varying polarities were used to analyze the TLC (Fluka, silica gel F_254_ plates) finger print profile of the extracts eluted in BEA (benzene:ethanol:ammonium hydroxide, 36:4:0.4), CEF (chloroform:ethyl acetate:formic acid, 20:16:4), and EMW (ethyl acetate:methanol:water, 20:10.8:8). Eluted plates were visualized under ultraviolet light at 254 and 360 nm and thereafter sprayed with freshly prepared vanillin spray reagent (0.1 g vanillin, 28 mL, methanol, 1 mL sulfuric acid) to visualize separated compounds. The plates were carefully heated at 110°C for optimal color development.^12^

### Phytoconstituents Analysis Test of Extracts

The following photochemical analysis were performed: reducing sugars and anthraquinones,^13^ saponins,^14^ tannins,^15^ alkaloids,^16^ cardiac glycosides (Keller- Killiani test), steroids, phlobatannin, terpenoids (Salkowski test), and flavonoids.^17^

#### Total Phenolic Content Determination

The concentration of phenolic content in 70% aqueous acetone extracts of the selected plants was determined using spectrophotometric method described by Singleton et al in 1999^18^ with modifications. The determination of the total phenol content employed the Folin-Ciocalteau method, where 0.1 mL of extract and 0.9 mL of distilled water were mixed in a 25 mL volumetric flask. To this mixture 0.1 mL of Folin-Ciocalteau phenol reagent was added and the mixture shaken well. One milliliter of 7% sodium carbonate (Na_2_CO_3_) solution was added to the mixture. After 5 minutes the volume was made up to 2.5 mL with distilled water. A set of standard solutions of gallic acid (0.0625, 0.125, 0.25, 0.5, and 1 mg/mL) were prepared as described above. The mixtures were incubated for 90 minutes at room temperature and the absorbance for test and standard solutions were determined against the reagent blank at 550 nm with an ultraviolet/visible spectrophotometer. Total phenol content was expressed as mg of gallic acid equivalents (GAE)/g of extract.^19^

#### Total Tannin Content Determination

The tannin content was determined using Folin-Ciocalteau method. About 0.1 mL of the 70% aqueous acetone extracts of the selected plants was added to a 10 mL volumetric flask with 5 mL of distilled water. To this mixture, 0.2 mL of 2 M Folin-Ciocalteau phenol reagent and 1 mL of 35% Na_2_CO_3_ solution was added and this was made up to 10 mL with distilled water. The mixture was shaken well and kept at room temperature for 30 minutes. A set of standard solutions of gallic acid (0.0625, 0.125, 0.25, 0.5, and 1 mg/mL) were prepared in the same manner as described above. Absorbance for test samples and standard solutions were measured against the blank at 725 nm with an ultraviolet/visible spectrophotometer. The tannin content was expressed as mg of GAE/g of extract.^19^

#### Total Flavonoid Content Determination

Total flavonoid content was determined by the aluminum chloride colorimetric assay. One milliliter of 70% aqueous acetone extracts of the plant was mixed with 4 mL of distilled water in a 10 mL volumetric flask. To the flask, 0.30 mL of 5% sodium nitrite was added. About 0.3 mL of 10% aluminum chloride was added to the mixture after 5 minutes, this was mixed. After 5 minutes, 2 mL of 1 M sodium hydroxide was added and this was made up to 10 mL with distilled water. A set of reference standard solutions of quercetin (0.0313, 0.0625, 0.125, 0.25, 0.5 mg/mL) were prepared in the same manner as described above. The absorbance for test and standard solutions were determined against the reagent blank at 510 nm with an ultraviolet/visible spectrophotometer. The total flavonoid content was expressed as mg of quercetin equivalents (QE)/g of extract.^19^

### Qualitative Antioxidant Activity

TLC plates were used to separate crude extracts as described earlier. To detect antioxidant activity, chromatograms were sprayed with 0.2% (w/v) 2,2-diphenyl-1-picrylhydrazyl (DPPH) (Sigma Chemicals) in methanol as an indicator. The presence of yellow zones against a purple background on chromatograms indicate the presence scavenging activity of free radicals by compounds present in the plant extracts.^20^

#### Reducing Power Assay

Antioxidant capacity as per reducing power assay was measured according to a method reported by Oyaizu.^21^ Briefly, a set of 5 dilutions of each plant extract (0.0625-1000 µg/mL) was prepared in 50% aqueous methanol. In a test tube, 2 mL plant extract, 2 mL sodium phosphate buffer (0.1 M, pH 6.6) and 2 mL potassium ferricyanide (1% w/v in distilled water) were added and mixed well. The mixture was incubated in a water bath for 20 minutes at 50°C. Then, 2 mL trichloroacetic acid (10% w/v in distilled water) was added and the mixture was centrifuged at 650 rpm for 10 minutes. The supernatant (3 mL) was taken into a test tube and 10 mL distilled water and 1 mL ferric chloride (0.1% w/v in distilled water) solution was added and mixed well. Absorbance was measured at 700 nm. Blank for each solvent was run using the same procedure but replacing the plant extract with an equal volume of solvent.

#### DPPH Free Radical Scavenging Activity

The DPPH free radical scavenging activity was determined according to method described by Brand-Williams et al.^22^ Briefly, serial dilution (0-1000 µg/mL) of the plant extract (1 mL) was mixed with 1 mL of 0.2% DPPH solution. A serial dilution of ascorbic acid in place of the extract was used as a standard from which the extracts were compared and absolute DPPH was used as a control (100% DPPH solution). The mixtures were incubated in the dark for about 30 minutes and afterward the absorbance was read at 517 nm. Linear regression plots derived from the tests were used to calculate the concentration exhibiting 50% of DPPH free radical scavenging activity (EC_50_).

### Antibacterial Activities

#### Test Microorganisms

Four bacteria species were obtained from the Department of Biochemistry, Microbiology, and Biotechnology, Faculty of Science and Agriculture, University of Limpopo. Two Gram-negative bacteria (*Escherichia coli* ATCC 25922 and *Pseudomonas aeruginosa* ATCC 27853) and 2 Gram-positive bacteria (*Enterococcus faecalis* ATCC 29212 and *Staphylococcus aureus* ATCC 29213) were maintained on nutrient agar slants. Organisms were subcultured in nutrient broth, incubated at 37°C for 24 hours and stored at 4°C as stock cultures. These organisms are mainly the strains recommended for use by the National Committee for Clinical Laboratory Standards.^23^

#### Bioautography

Thin layer chromatographic plates spotted with 20 µL of each extract (10 mg/mL) were eluted in solvent systems as previously described without spraying with vanillin reagent and dried under a stream of cold air for 5 days to allow evaporation of eluent solvents. The chromatograms were sprayed with overnight culture of the bacterial species until completely wet and incubated at 37°C in a humidified chamber for 24 hours. The plates were sprayed with 2 mg/mL of *p*-iodonitrotetrazolium violet (INT) (Sigma Chemicals) and further incubated for 2 hours. White areas against pink background indicate where reduction of INT to the colored formazan did not take place due to the presence of compounds that inhibited the growth of tested bacteria.^24^

#### Minimum Inhibitory Concentration

The minimum inhibitory concentration (MIC) value of plant extracts was determined using the method as described by Eloff.^25^ The plant extracts (10 mg/mL) were serially diluted 50% with sterile water in 96-well plates. One hundred microliter of overnight bacterial culture was transferred into each well and acetone was included as a solvent control. The microtiter plates were incubated at 37°C for 24 hours. After incubation, 20 µl of 2 mg/ml of *p*-iodonitrotetrazolium violet (INT) (Sigma) was added to each well as an indicator of growth. The plates were further incubated for 30 minutes and all determinations were carried out in triplicate. Growth of microorganism was indicated by the emergence of a purple-red color resulting from the reduction of INT into formazan. The MIC was recorded as the lowest concentration of the extract that inhibited bacteria growth after 24 hours of exposure of the extracts.

### Statistical Analysis

All the determinations were conducted at least 3 times (*n* = 3); the statistical mean was calculated with ±SD using Microsoft Excel 2013.

## Results and Discussion

**Figure 1.**
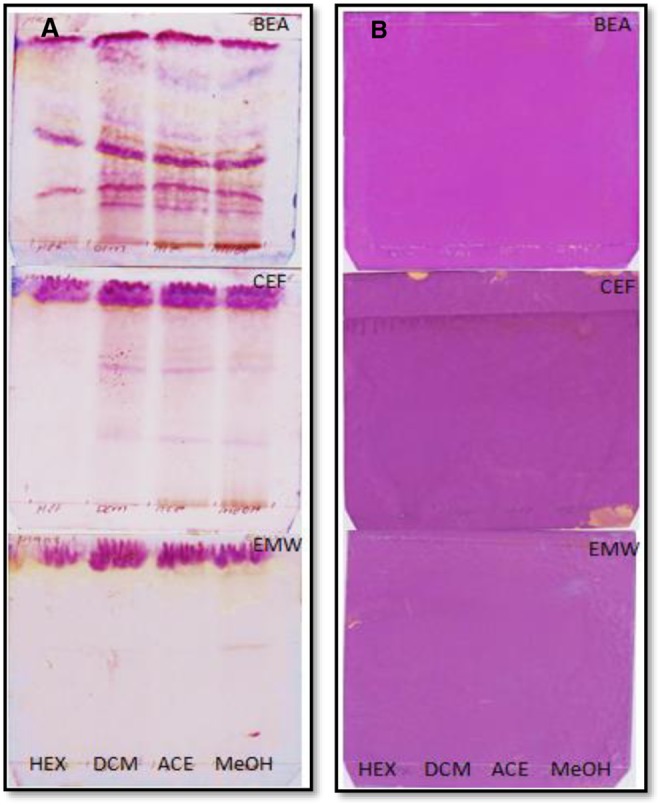
Chromatograms of *Spilanthes mauritiana* crude extracts developed in 3 solvents systems: BEA, CEF, and EMW from top to bottom, sprayed with vanillin sulfuric acid reagents (A) and 0.2% DPPH in methanol as an indicator (B). Compounds were extracted with hexane (HEX), dichloromethane (DCM), acetone (ACE), and methanol (MeOH) in lanes from left to right. BEA, benzene:ethanol:ammonium hydroxide, 36:4:0.4; CEF, chloroform:ethyl acetate:formic acid, 20:16:4; EMW, ethyl acetate:methanol:water, 20:10.8:8; DPPH, 2,2-diphenyl-1-picrylhydrazyl.

Extraction of phytochemical compounds from medicinal plants is highly dependent on the type and polarity of solvent used.^26^ Hexane, dichloromethane, acetone, and methanol were used to extract phytochemical compounds from *S mauritania.* Methanol extracted the highest mass of extract (2.95%), followed by hexane (2.8%), acetone (1.05%), and dichloromethane (0.14%) in that order (Table 1). Extracts were reconstituted in acetone to the final concentration of 10 mg/mL for determination of their TLC fingerprint profile in 3 eluent systems of varying polarities namely BEA, CEF, and EMW. Chromatograms were sprayed with vanillin sulfuric acid to show phytochemical compounds present in the different extracts (Figure 1a). BEA solvent system is shown to best separate the constituents in extracts with more bands followed by CEF and EMW in that order.

**Table 1.** Percentage Yield Extracted From *Spilanthes mauritiana* With Solvents of Varying Polarities.

Solvents	%
Hexane	2.8
Dichloromethane	0.14
Acetone	1.05
Methanol	2.95

**Figure 2.**
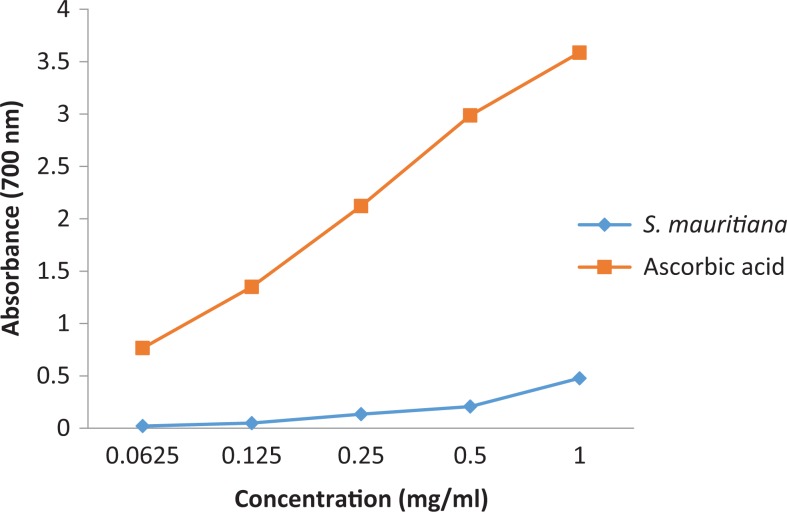
Reducing power assay of *Spilanthes mauritiana* extract expressed as absorbance at 700 nm (n = 3).

To detect the presence of antioxidant constituents, chromatograms were sprayed with 0.2% DPPH in methanol as an indicator (Figure 1b). Constituents in extracts are shown to exhibit minimal antioxidant potential on TLC in the DPPH free radical scavenging assay, possibly due to the small amount of compound with antioxidant activity contained in the extracts, which are very polar in nature. From the literature, acetylenes, spilanthol, and related compounds have been isolated from the leaves of *S mauritiana*.^8^ Spilanthol-rich extracts from flowers of *Spilanthes acmella* in a study by Dias et al^27^ have been shown to present high antioxidant and anti-inflammatory activities. Although different plant part is used in this study, the possibility that this compound maybe responsible for the observed antioxidant activity may not be rule out. This may possibly explain low amount of the antioxidant constituent detected. Reducing power of the acetone extract of *S mauritiana* was determined and the results are shown in Figure 2.

The extract displayed low reducing power, which explain low antioxidant activity on the TLC sprayed with DPPH. Although the extract had low reducing power, it showed on increasing trend in reducing power with the increase in extract concentration. In this assay, the presence of reducers (ie, antioxidants) causes the reduction of the Fe^3+^/ferricyanide complex to the ferrous form. Therefore, measuring the formation of Perl’s Prussian blue at 700 nm can monitor the Fe^2+^ concentration.^28^ The examination of antioxidant activity of plant extract was further quantified and it had DPPH scavenging potential EC_50_ of 0.74 ± 0.0014, which clarify less activity on TLC plates.

**Figure 3.**
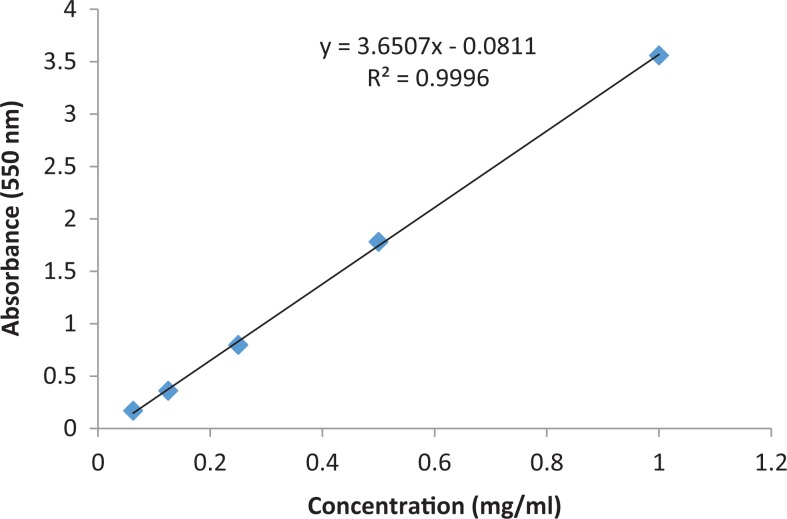
Calibration graph for total phenolic content.

**Figure 4.**
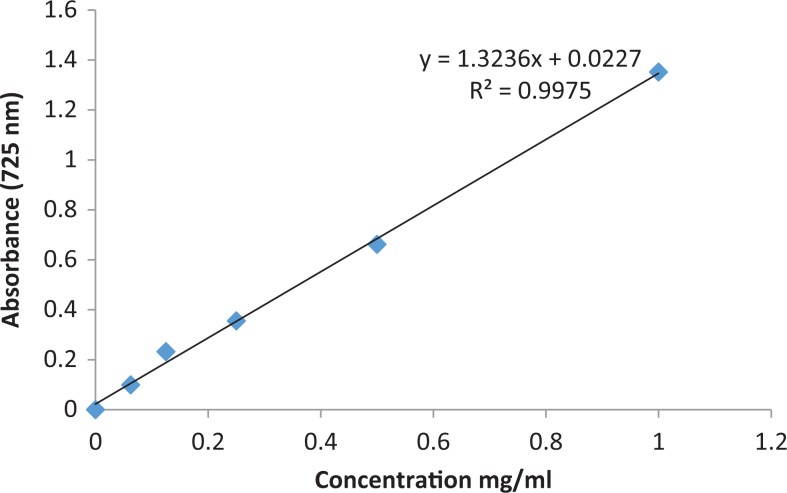
Calibration graph for tannin content.

**Figure 5.**
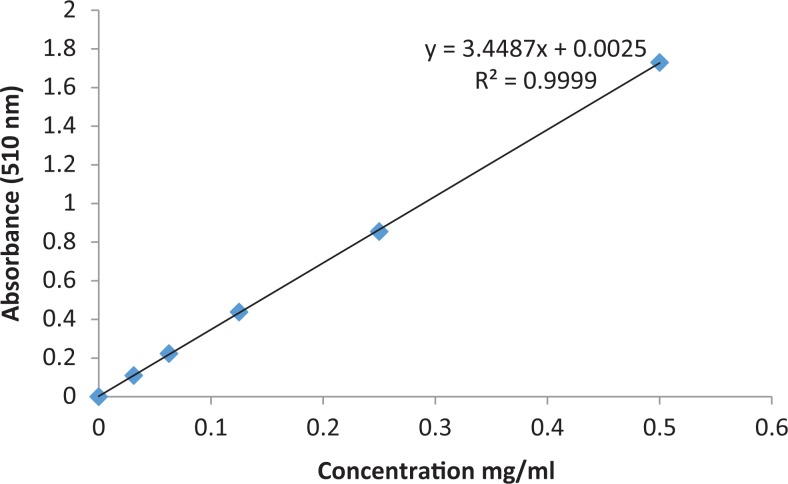
Calibration graph for total flavonoid content.

Table 2 shows the presence of flavonoids, reducing sugar, steroids, tannins, and terpenoids and alkaloids and absence of anthraquinones, cardiac glycosides, phlobatanin, and saponins. Extract was prepared to examine the total phenolic content, tannin, and flavonoid. The results for extractive value and for total phenol, tannin, and flavonoid contents are tabulated in Table 3. Calibration graphs for total phenol, tannin, and flavonoid contents are shown in Figures 3, 4, and 5, respectively. The total phenolic contents in the examined plant extracts using the Folin-Ciocalteu’s reagent is expressed in terms of GAE (the standard curve equation: *y* = 3.6507*x* – 0.0811, *R* ^2^ = 0.9996). The total phenolic contents in the examined acetone extract was 52.47 ± 2.29 mg GAE/g, which was high.^29^

**Table 2.** Phytochemical Constituents of *Spilanthes mauritiana* Leaves.

Constituents	Occurrence^a^
Terpenes/Terpenoids	+
Tannins	+
Steroids	+
Reducing sugar	+
Saponins	−
Phlobatannin	−
Alkaloids	+
Flavonoids	+
Anthraquinones	−
Cardiac glycosides	−

^a^“+” indicates present and “−” indicates absent.

**Table 3.** Total Phenolic Content, Tannin, Flavonoid, and DPPH Free Radical Scavenging Activity (EC_50_) of the *Spilanthes mauritiana* Leaves.^a^

*Spilanthes mauritiana* Leaves	Total Phenols (mg of GAE/g of Sample)	Tannins (mg of GAE/g of Sample	Flavonoids (mg of QE/mg of Sample)	DPPH Scavenging Potential EC_50_
Extract	52.47 ± 2.29	23.9 ± 1.18	25.1 ± 0.79	0.74 ± 0.0014
Ascorbic acid				0.10 ± 0.0014

Abbreviations: DPPH, 2,2-diphenyl-1-picrylhydrazyl; GAE, gallic acid equivalent; QE, quercetin equivalent.

^a^Each value is the average of 3 analyses ±standard deviation.

The tannin content was also examined in acetone extract using the Folin-Ciocalteu’s reagent, expressed in terms of GAE (the standard curve equation: *y* = 1.3236*x* + 0.0227, *R* ^2^ = 0.9975). The tannin concentration measured was 23.9 ± 1.18 mg of GAE/g (Table 3). The concentration of flavonoids in *S mauritiana* was determined using spectrophotometric method with aluminum chloride. The content of flavonoids was expressed in terms of QE (the standard curve equation: *y* = 3.4487*x* + 0.0025, *R* ^2^ = 0.999). The extract of *S mauritiana* contains the moderate flavonoid concentration of 25.1 + 0.79 mg QE/g (Table 3).

**Figure 6.**
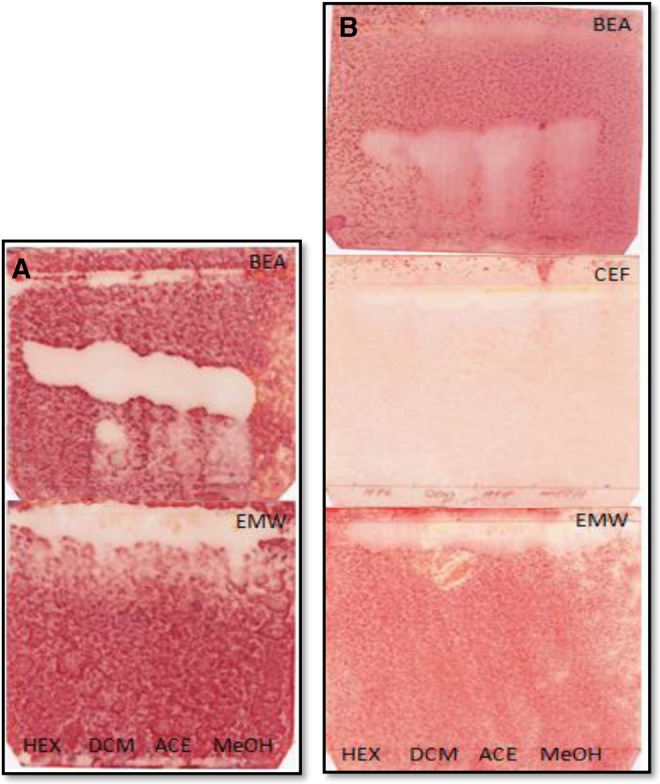
Bioautograms of *Spilanthes mauritiana* crude extracts separated with 3 solvent systems: BEA, CEF, and EMW and sprayed with *Escherichia coli* (A), and *Pseudomonas aeruginosa* (B), white zones indicate active compounds that inhibited growth of tested bacterial species. Hexane (HEX), dichloromethane (DCM), acetone (ACE), and methanol (MeOH) in lanes from left to right. BEA, benzene:ethanol:ammonium hydroxide, 36:4:0.4; CEF, chloroform:ethyl acetate:formic acid, 20:16:4; EMW, ethyl acetate:methanol:water, 20:10.8:8.

**Figure 7.**
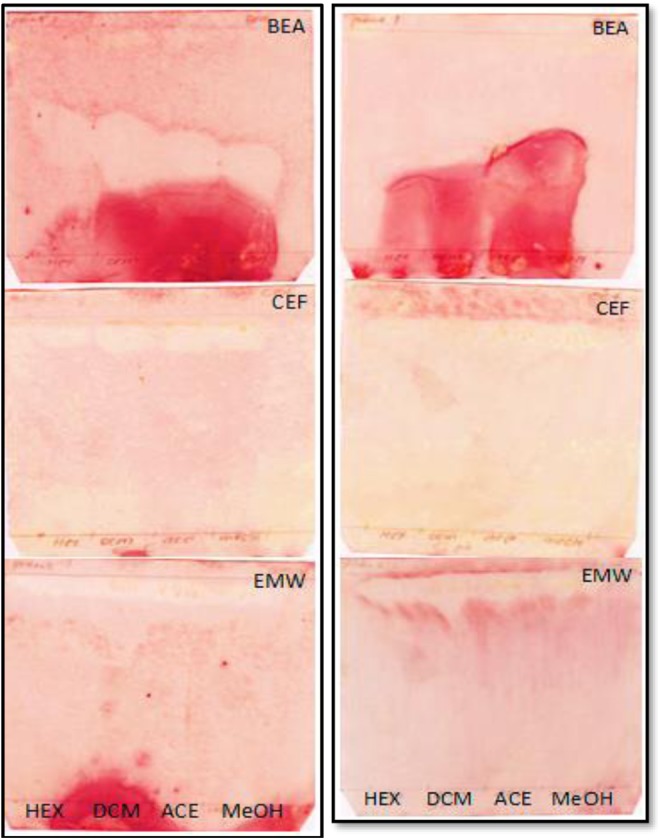
Bioautograms of *Spilanthes mauritiana* crude extracts separated with 3 solvent systems: BEA, CEF, and EMW and sprayed with *Enterococcus faecalis* (A) and *Staphylococcus aureus* (B), white zones indicate active compounds that inhibited growth of tested bacterial species. Hexane (HEX), Dichloromethane (DCM), acetone (ACE) and methanol (MeOH) in lanes from left to right. BEA, benzene:ethanol:ammonium hydroxide, 36:4:0.4; CEF, chloroform:ethyl acetate:formic acid, 20:16:4; EMW, ethyl acetate:methanol:water, 20:10.8:8.

*S mauritiana* is commonly used as a chewing stick to cure tonsillitis and other bacterial-related diseases. The ethanol and aqueous extracts have been found not to be effective against *S pyogenes* a causative agent of tonsillitis.^7^ However, *Spilanthes acmella*, a member of the Asteraceae family has been reported to have multiple pharmacological actions, which includes antifungal, antipyretic, anesthetic, insecticidal, anticonvulsant, antioxidant, aphrodisiac, analgesic, as a pancreatic lipase inhibitor, antimicrobial, antinociception, diuretic, vasorelaxant, anti-human immunodeficiency virus inhibitor, for toothache relieve and anti-inflammatory effects.^30^ In the present study, antibacterial activity of *S mauritiana* extracts were evaluated against Gram-negative and Gram-positive bacteria using bioautography and microbroth dilution methods. White spots against pink background on bioautograms indicate the presence of antibacterial compounds active against tested organism. Chromatograms were separated in 3 mobile phases and sprayed with *E coli* (Figure 6a), *P aeruginosa* (Figure 6b), *Enterococcus faecalis* (Figure 7a) and *S aureus* (Figure 7b). BEA separation system showed the presence of potent antibacterial compounds in all the extracts with R_f_ value of 0.50 against *E coli*, *P aeruginosa* (0.56), *E faecalis* (0.43), and *S aureus* (0.44). MICs of tested organisms were also calculated (Table 4). Ampicillin was used as a positive control.

**Table 4.** Minimum Inhibitory Concentration (MIC) Values (mg/mL) of Various Extracts of *Spilanthes mauritiana* Crude Extracts Against 4 Tested Bacterial Species.

	MIC Values (mg/mL)				
Organisms	HEX	DCM	ACE	MeOH	Ampicillin
*Esherichia coli*	2.50	2.50	2.50	1.25	0.16
*Pseudomonas aeruginosa*	−	2.50	2.50	2.50	0.12
*Enterococcus faecalis*	2.50	0.32	0.32	0.63	0.16
*Staphylococcus aureus*	−	2.50	2.50	2.50	0.04

Abbreviations: HEX, hexane; DCM, dichloromethane; ACE, acetone; MeOH, methanol; “**–**” no activity.

According to Eloff,^11^ acetone has been tested and reported to dissolve many of hydrophilic and lipophilic components, is also miscible with water and less toxic to bacteria. High inhibitory activity of extracts is indicated by the lowest concentration of extracts (low MIC value) that inhibited bacterial growth. In the present study, MIC values of all plant extracts were too high. Hexane extract had an average MIC value of 2.50 mg/mL, followed by methanol extract (1.72 mg/mL), acetone and dichloromethane extracts (1.96 mg/mL) (Table 4). *E faecalis* was shown to be the most sensitive organism to the effect of the dichloromethane, acetone, and methanol extracts with an MIC values of 0.32 mg/mL. A related plant in the same family (*S mauritiana*) have been reported by Ferreira de Lima et al^31^ to have MIC values between 130 and 8000 μg/mL against *S aureus*, *Bacillus cereus*, *Bacillus subtilis*, *E coli*, and *P aeruginosa*. Extracts from the roots and flower heads of *S mauritiana* in a preliminary study have been reported to possess antibacterial activity.^32,33^ The present study is consistent with previous reports on the antibacterial activity of *S mauritiana* against tested microorganisms. Total activity of a plant extract is defined as the amounts of material extracted from a single gram of dried plant material divided by the MIC value. Total activity is important when evaluating the potential use of a plant extract for treating fungal and bacterial infections.^34^ Methanol extract had the highest average total activity of 23.51 mg/L followed by acetone (11.35 mg/L), hexane extract (11.2 mg/L), and dichloromethane (1.52 mg/L) the lowest (Table 5). Taken together, MIC values obtained in the study suggests the plant to contain bioactive compounds active against tested pathogen that can be used either as single entities or templates for the treatment of bacterial infections.

**Table 5.** Total Activity (mL/g) of *Spilanthes mauritiana* Crude Extracts Against 4 Bacterial Test Organisms.

	Total Activity (mL/g)			
Organisms	HEX	DCM	ACE	MeOH
*Escherichia coli*	11.2	0.56	4.20	23.60
*Pseudomonas aeruginosa*	**–**	0.56	4.20	11.8
*Enterococcus faecalis*	11.2	4.38	32.81	46.83
*Staphylococcus aureus*	**–**	0.56	4.20	11.8
Average	11.2	1.52	11.35	23.51

Abbreviations: HEX, hexane; DCM, dichloromethane; ACE, acetone; MeOH, methanol; “**–**” no activity.

## Conclusion

The present study reveals antibacterial activity of *S mauritiana* crude extracts against tested microorganisms. The activities of most of the plant extracts were higher than that of the positive controls expect *E faecalis*, which was most sensitive. It will be reasonable therefore to consider the extracts for isolation of active compounds. Potent antibacterial compounds were shown to be present in all tested extract on bioautograms with minimal antioxidant constituents of high polarity. The study serves as a scientific proof for the use of this plant in traditional medicine. Furthermore, the study also provides valuable information for further phytochemical isolation and characterization studies of active compounds, with potential for the development of new drugs.
